# Diagnostic Accuracy of 3 Mpox Lateral Flow Assays for Antigen Detection, Democratic Republic of the Congo and United Kingdom

**DOI:** 10.3201/eid3106.250166

**Published:** 2025-06

**Authors:** Elie Ishara-Nshombo, Anushri Somasundaran, Alessandra Romero-Ramirez, Konstantina Kontogianni, Daniel Mukadi-Bamuleka, Marithé Mukoka-Ntumba, Emile Muhindo-Milonde, Hugues Mirimo-Nguee, Jacob Parkes, Yusra Hussain, Susan Gould, Christopher T. Williams, Dominic Wooding, Juvenal Nkeramahame, Mikaela Watson, Hayley E. Hardwick, Malcolm G. Semple, John Kenneth Baillie, Jake Dunning, ISARIC 4, C Investigators,, Thomas E. Fletcher, Thomas Edwards, Devy M. Emperador, Hugo Kavunga-Membo, Ana Isabel Cubas-Atienzar

**Affiliations:** Rodolphe Mérieux Laboratory, Institut National de Recherche Biomédicale, Kinshasa, Democratic Republic of the Congo (E. Ishara-Nshombo, D. Mukadi-Bamuleka, M. Mukoka-Ntumba, E. Muhindo-Milonde, H. Mirimo-Nguee, H. Kavunga-Membo); Liverpool School of Tropical Medicine, Liverpool, UK (A. Somasundaran, A. Romero-Ramirez, K. Kontogianni, J. Parkes, Y. Hussain, S. Gould, C.T. Williams, D. Wooding, T.E. Fletcher, T. Edwards, A. Cubas-Atienzar); Royal Liverpool University Hospital NHS Foundation Trust, Liverpool (S. Gould, T.E. Fletcher); Foundation for Innovative New Diagnostics, Geneva, Switzerland (J. Nkeramahame, M. Watson, D.M. Emperador); University of Liverpool, Liverpool (H.E. Hardwick, M.G. Semple); University of Edinburgh, Edinburgh, Scotland, UK (J.K. Baillie); Pandemic Sciences Institute, University of Oxford, Oxford, UK (J. Dunning); Royal Free London NHS Foundation Trust, London, UK (J. Dunning)

**Keywords:** mpox, monkeypox virus, MPXV, sexually transmitted infections, Orthopoxvirus, Poxviridae, point-of-care test, POCT, diagnostic, evaluation, lateral flow assay, LFA, lateral flow test, LFT, rapid diagnostic test, RDT, Democratic Republic of the Congo, United Kingdom

## Abstract

The ongoing outbreaks of mpox highlight the urgent need for a rapid and low-cost diagnostic test to accurately detect and control this emerging disease. We estimated the analytical sensitivity using viral culture of the monkeypox virus clade IIb lineage B1 and clinical diagnostic performance of 3 antigen detection rapid diagnostic tests (Ag-RDT) by using skin swab samples and upper-respiratory swab samples from mpox patients in the Democratic Republic of the Congo and the United Kingdom. The analytical limit of detection was 1.0 × 10^4^ plaque-forming units/mL, fulfilling World Health Organization recommendations. Specificity of the 3 Ag-RDTs was 100%, but sensitivity was estimated at 0.00%–15.79% using skin samples and 0.00% using respiratory samples. None of the 3 Ag-RDTs reached the World Health Organization’s target clinical sensitivity, and we do not recommend them as diagnostic or screening tools for suspected mpox cases. Accurate Ag-RDTs for mpox diagnosis remain urgently needed.

Mpox is a zoonotic disease caused by monkeypox virus (MPXV), which belongs to the *Orthopoxvirus* genus, Poxviridae family; it has 2 major clades: clade I, which is subdivided into subclade Ia and Ib; and clade II, which is subdivided into subclade IIa and IIb (*1*). Historically, mpox was endemic to Central and West Africa. In May 2022, the number of mpox cases increased in a surge that included rapid expansion in nonendemic countries; it was declared the first mpox public health emergency of international concern (PHEIC) by the World Health Organization (WHO) (*2*). Since then, >100,000 cases of mpox and >200 deaths have been described in >120 countries not previously considered mpox endemic. The number of infections during the 20th Century has already been surpassed by the number of cases that occurred after the 2022 outbreak caused by clade II (*3*). On August 14, 2024, a second mpox PHEIC was declared by WHO after the substantial increase in mpox cases in the Democratic Republic of the Congo (DRC) and neighboring countries (*4*). In 2024, DRC, where mpox was first identified in 1970, reported the highest number of suspected cases globally, >27,000, and 800 deaths (*5*). 

In the United Kingdom, most cases before 2022 were associated with travel from mpox-endemic countries. During August 2018–September 2021, a total of 7 mpox cases were identified in the United Kingdom (4 imported cases and 3 secondary cases) (*6*). The discovery of the first mpox case of the 2022 global outbreak in the United Kingdom was on May 7, 2022, in a person who traveled from Nigeria; as of June 8, 2022, a total of 336 cases had been laboratory confirmed, a number that rose to 3,732 by the end of 2022. On October 30, 2024, the first clade Ib mpox case was confirmed in the United Kingdom; 3 further household contact cases were confirmed (*7*). Those were the first locally transmitted clade Ib mpox cases in the WHO European Region and the first outside Africa since a PHEIC was declared for a second time in August 2024 (*4*).

To confirm a clinical diagnosis, WHO advises testing for MPXV as soon as possible in persons who fit the suspected case definition. Laboratory-based real-time PCR is the primary method used for MPXV detection. Laboratory-based PCR testing requires costly equipment, up-front DNA extraction, and skilled personnel, which might only be available in specialized laboratories, making rapid detection of cases during outbreaks more challenging. In contrast, rapid diagnostic tests (RDTs) are low cost, equipment free, easy to use, and suitable to use at the point of care (POC); results are available within 20 minutes (*8*). The value of antigen-detecting RDTs (Ag-RDTs) in rapidly detecting infected persons and enabling isolation and management of patients has been proven for many viral diseases, notably during the COVID-19 pandemic (*9*).

The global increase in mpox cases after the 2022 PHEIC and the subsequent PHEIC 15 months later brought to light the increased demand for decentralized POC diagnostics for this highly infectious virus and highlighted the urgent need for Ag-RDTs for MPXV as a priority. This increased demand has resulted in the availability of dozens of Ag-RDTs in the market. As of January 2024, >69 Ag-RDTs for MPXV were in varying stages of development, of which >35 had received CE marking for in vitro diagnostics (IVD) (required for devices sold in Europe) and were commercially available (*10*). Despite the increased number of Ag-RDTs for MPXV, clinical evaluation data are still lacking (*11*). To ensure reliable and accurate performance of Ag-RDTs, diagnostic evaluation studies across multiple, independent sites are required to generate evidence of their effectiveness to guide implementation.

The aim of this study was to evaluate the diagnostic performance of 3 Ag-RDT brands at detecting MPXV antigens: FlowFlex Monkeypox Virus Antigen Rapid Test (ACON Biotech [Hangzhou] Co., Ltd., https://www.aconbio.com), Ecotest Monkeypox Antigen Rapid Test (Assure Tech [Hangzhou] Co., Ltd., https://www.assuretech-product.com), and Standard Q Monkeypox Ag Test (SD Biosensor, https://www.sdbiosensor.com). We used skin lesion swab samples and upper respiratory tract swab samples from patients in DRC and the United Kingdom, 2 countries with different MPXV epidemiologic characteristics and clades (clade I [DRC] and clade II [United Kingdom]). We evaluated the Ag-RDTs in prospectively collected samples in DRC and retrospectively in the United Kingdom.

## Materials and Methods

### Study Settings and Participants

In DRC, persons >2 years of age suspected to have mpox according to the WHO case definition (*12*) were eligible to participate in the study. Ethical approval was obtained by DRC’s National Ethics and Health Committee (Comité National d’Ethique et de la Santé [CNES], reference 452/CNES/BN/PMMF/2023). Recruitment took place during January–December 2023 in Maniema Province through home visits of eligible persons. Paired skin samples and upper respiratory specimens were collected from all recruited participants (n = 68) by trained heathcare workers and placed in 3 mL of noninactivating virus transport medium (VTM) for viral preservation. The Ag-RDTs could not be tested at the POC because of health and safety restrictions; all VTM samples were transported in cool boxes (2°C–8°C) to the Institut National de Recherche Biomédicale (INRB) Biosafety Level 2 laboratories in Lubutu for processing according to national guidance for MPXV testing. All VTM tubes were processed within 4 hours for MPXV Ag-RDT testing and quantitative PCR (qPCR).

In the United Kingdom, we used retrospectively collected skin samples (n = 30) and upper respiratory samples (n = 23 [1 nasopharyngeal, 22 oropharyngeal]) in universal transport media (UTM) (Copan, https://www.copangroup.com) from a cohort of 16 adult patients positive for mpox by PCR from the Royal Liverpool University Hospital, Sheffield Teaching Hospital NHS Foundation Trust, and Royal Free London Hospital for this study. Patients were recruited during the last 2 outbreaks of mpox in the United Kingdom in 2018 and 2022. Trained healthcare workers collected all swab samples. Patients gave consent under the WHO ISARIC Clinical Characterization Collaboration Protocol for severe emerging infections (ISRCTN66726260). Ethical approval was obtained from the National Research Ethics Service and the Health Research Authority (IRAS ID:126600, REC 13/SC/0149). 

Mpox diagnosis was confirmed by the UK Health Security Agency using qPCR before enrollment in the study. In addition to samples from mpox-positive patients, to fulfill the minimum number of negative swab specimens for mpox diagnostic evaluations recommended by the US Food and Drug Administration (*13*), we used a set of 32 leftover nasopharyngeal samples in UTM from previous COVID-19 studies (*14*) as mpox negative controls. The samples were collected under the Facilitating Accelerated Clinical Validation of Novel diagnostics for COVID-19 (FALCON), and ethical approval was obtained from the National Research Ethics Service and the Health Research Authority (IRAS ID:28422, REC: 121 20/WA/0169). All samples were aliquots stored at –80°C and thawed for the first time for this study. Samples were processed and tested at the Biosafety Level 3 laboratories of the Liverpool School of Tropical Medicine (LSTM) as previously described (*14*).

### MPXV Ag-RDT Testing

We selected the Ag-RDTs evaluated in this study after an expression of interest launched by FIND (https://www.finddx.org) and a scoring process based on defined criteria. We evaluated 3 Ag-RDTs: FlowFlex Monkeypox Virus Antigen Rapid Test, Ecotest Monkeypox Antigen Rapid Test, and Standard Q Monkeypox Ag Test. The 3 RDTs are based on immunochromatography and show the presence of MPXV A29L antigen using colloidal gold for visualization. Flowflex and Ecotest were commercially available, whereas Standard Q was for research use only at the time of evaluation. All test brands can be used with skin lesion samples. In addition, Flowflex can be used with serum, plasma, and upper respiratory samples; Standard Q can be used in serum, plasma, and whole-blood samples; and Ecotest can be used in upper respiratory samples.

We performed tests in INRB Biosafety Level 2 laboratories in DRC and in LSTM Biosafety Level 3 laboratories in the United Kingdom. In brief, we added the specified amount of VTM or UTM confirmed by the manufacturers (200 μL for Flowflex and Ecotest and 300 μL for Standard Q) into the extraction buffer and then added the number of drops of the extraction buffer specified in the instructions for use into the sample well (4 drops for Flowflex and Standard Q and 3 drops for Ecotest). We read tests and interpreted them visually after 15–30 minutes according to the instructions. Two independent technicians read the results; a third technician acted as a tiebreaker in case of discrepant results.

### Reference MPXV qPCR Test

At both sites, we extracted DNA and performed MPXV qPCR using the same UTM or VTM tube used for the 3 Ag-RDT tests. At INRB, we extracted DNA from a 300-μL aliquot of sample by using the Natch 16S automated platform with the Nucleic Acid Extraction-Purification Kit (both Sansure Biotech, https://www.sansureglobal.com), according to the instructions for use. At LSTM, we extracted DNA from 200 μL of UTM using the QiAamp96 Virus Qiacube HT kit (QIAGEN, https://www.qiagen.com), according to the instructions for use.

We used the same MPXV qPCR reference test in both sites for evaluating index tests (Monkeypox Virus Nucleic Acid Diagnostic Kit; Sansure Biotech). We carried out qPCR by using a MA-1620Q qPCR thermocycler (Sansure Biotech) at INRB and a QuantStudio 5 (Thermo Fisher Scientific, https://www.thermofisher.com) at LSTM. We considered a qPCR result with a cycle threshold (Ct) <40 MPXV positive according to instructions for use. We used this qPCR kit as the reference test because it has been successfully demonstrated to detect MPXV clades I, IIa, and IIb (*15*), is CE marked for commercial use, and has demonstrated higher diagnostic accuracy than the mpox Centers for Disease Control and Prevention laboratory–based qPCR (*16*).

### Analytical Limit of Detection of Ag-RDTs

We cultured mpox viral stock of a MPXV strain from clade II, subclade IIb, lineage B.1 (Slovenia_MPXV–1_2022) obtained from the European Virus Archive Global (https://www.european-virus-archive.com) in Vero E6 cells (ECACC 85020206) in Dulbecco’s Modified Eagle Medium plus 10% fetal bovine serum and 1% penicillin/streptomycin solution to generate the MPXV stock. We serially diluted a fresh aliquot 10-fold using UTM to produce concentrations from 5.0 × 10^4^ to 5.0 × 10^0^ PFU/mL. We defined the limit of detection (LOD) as the lowest concentration at which all 3 replicates were positive. Once the LOD was achieved, half dilutions were tested above and below the LOD. We performed Ag-RDT testing to calculate the LOD and quantified the viral copy numbers per mL (copies/mL) of the serial dilutions, as previously described (*14*,*16*).

### Statistical Analysis

To assess the diagnostic accuracy of Ag-RDTs in patients with suspected mpox, we calculated point estimates of sensitivity and specificity for each Ag-RDT on the basis of results of the reference MPXV qPCR assay from the same VTM or UTM tube used for the Ag-RDT. We derived the 95% CI for each point estimate on the basis of Wilson’s score method. To compare performance of the Ag-RDTs at different Ct values, we stratified point estimates of sensitivity by Ct value of the reference test. We used 2-tailed Fisher exact test and χ^2^ test to determine nonrandom associations between categorical variables. We assessed differences between the Ct values (expressed as mean +SD) in sample groups using the paired Student *t* test. Statistical significance was set at <0.05. We performed statistical analysis using R version 4.5.0 (R Foundation for Statistical Computing, https://ww.r-project.org) and GraphPad Prism version 9.1.0 (GraphPad Software, Inc., https://www.graphpad.com).

## Results

### Clinical Evaluation

 In DRC, 34/68 (50%) of mpox patients were men (Table 1). The median time from onset of symptoms was 4 (range 1–34) days. The most prevalent symptoms were fever (91%), skin lesions (100%), influenza-like symptoms (75%), headaches (54%), and cough (50%). In the United Kingdom, 16/16 (100%) mpox patients were men; mean age was 35.1 (range 24–58) years. The median time from onset of symptoms was 8 (range 0–11) days. The most common symptoms were skin lesions (100%), skin rashes (87.5%), and fever (68.8%).

**Table 1:** Clinical characteristics of recruited mpox patients in study of diagnostic accuracy of 3 mpox lateral flow assays for antigen detection, Democratic Republic of the Congo and United Kingdom*

Characteristic	Democratic Republic of the Congo, n = 68	United Kingdom, n = 16
Mean age (range), y	17 (2–47)	35.1 (24–58)
Sex		
M	34 (50)	16 (100)
F	34 (50)	0
Time from symptom onset, d		
Median (interquartile range)	4 (3–7)	8 (4.25–12.75)
0–3	32 (47)	1 (6.25)
4–7	22 (32)	6 (37.5)
>8	14 (21)	9 (56.25)
Symptoms		
Skin lesions	68 (100)	16 (100)
Fever	62 (91)	11 (68.75)
Influenza-like symptoms	51 (75)	4 (25)
Skin rashes	0	14 (87.5)
Headache	37 (54)	4 (25)
Cough	34 (50)	1 (6.25)
Sore throat	25 (37)	4 (25)
Nausea	20 (29)	0
Abdominal pain	19 (28)	0
Chest pain	14 (21)	0
Vomiting	6 (9)	0
Diarrhea	5 (7)	1 (6.25)
Painful Urination	4 (6)	0
Eye discharge	3 (4)	0
Redness of eyes	2 (3)	0

*Values are no. (%) except as indicated.

In DRC, 19/68 (28%) skin samples and 14/68 (21%) upper-respiratory samples from persons suspected of having mpox tested positive using the Sansure qPCR. Flowflex and Ecotest Ag-RDTs detected MPXV antigens in 3/19 MPXV-positive skin samples, resulting in a clinical sensitivity of 15.79% (95% CI 5.52%–37.57%), whereas Standard Q detected MPXV antigens in 2/19 samples, resulting in a clinical sensitivity of 10.53% (95% CI 2.94%–31.39%). The Ag-RDT Flowflex was more sensitive when detecting MPXV antigen in skin samples with Ct <20 than those with Ct values >33 (p = 0.008); however, this difference was not observed with the other Ag-RDT brands. None of the Ag-RDT brands detected MPXV antigen in upper respiratory samples, resulting in 0% (95% CI 0%–23.2%) sensitivity. The clinical specificity was 100% (95% CI 92.73%–100%) for each of the Ag-RDTs in both sample types (Table 2).

**Table 2:** Clinical diagnostic accuracy parameters of 3 MPXV antigen-detecting rapid diagnostic tests from 68 suspected mpox case-patients, the Democratic Republic of the Congo*

Category	Skin lesion swab samples, n = 68				Upper respiratory tract swab samples, n = 68		
	Ecotest	Flowflex	Standard Q		Ecotest	Flowflex	Standard Q
True positive	3	3	2		0	0	0
True negative	49	49	49		54	54	54
False positive	0	0	0		0	0	0
False negative	16	16	17		14	14	14
Specificity, % (95% CI)	100 (92.7–100)	100 (92.7–100)	100 (92.7–100)		100 (93.4–100)	100 (93.4–100)	100 (93.4–100)
Sensitivity, % (95% CI)	15.79 (5.5–37.6)	15.79 (5.5–37.6)	10.53 (2.9–31.4)		0 (0.0–23.2)	0 (0.0–23.2)	0 (0.0–23.2)
PPV, % (95% CI)	100 (29.2–100)	100 (29.2–100)	100 (15.8–100)		NA	NA	NA
NPV, % (95% CI)	75.38 (71.6–78.8)	75.38 (71.6–78.8)	74.24 (71.2–77.1)		79.41 (68.4–87.3)	79.41 (68.4–87.3)	79.41 (68.4–87.3)
Sensitivity by Ct, % (95% CI)							
Ct <20	0 (0.0–79.4), n = 1	100 (20.7–100), n = 1	0 (0.0–79.4), n = 1		NA	NA	NA
Ct <25	14.29 (2.6–51.3), n = 7	28.57 (8.2–64.1), n = 7	14.29 (2.6–51.3), n = 7		0 (0.0–79.4), n = 1	0 (0.0–79.4), n = 1	0 (0.0–79.4), n = 1
Ct <33	27.27 (9.8–56.7), n = 11	27.27 (9.8–56.7), n = 11	18.18 (5.1–47.7), n = 11		0 (0.0–39.0), n = 6	0 (0.0–39.0), n = 6	0 (0.0–39.0), n = 6
Ct <40	15.79 (5.5–37.6), n = 19	15.79 (5.5–37.6), n = 19	10.53 (2.9–31.4), n = 19		0 (0.0–21.5), n = 14	0 (0.0–21.5), n = 14	0 (0.0–21.5), n = 14

*Tests evaluated: Ecotest, Ecotest Monkeypox Antigen Rapid Test (Assure Tech [Hangzhou] Co., Ltd., https://www.assuretech-product.com); Flowflex, FlowFlex Monkeypox Virus Antigen Rapid Test (ACON Biotech [Hangzhou] Co., Ltd., https://www.aconbio.com); Standard Q, Standard Q Monkeypox Ag Test (SD Biosensor, https://www.sdbiosensor.com). Ct, cycle threshold; MPXV, monkeypox virus; NA, not available; NPV, negative predictive value; PPV, positive predictive value.

In the United Kingdom, 16/23 upper-respiratory samples (69.56%) and 27/30 skin samples (90%) from mpox-positive patients were positive by Sansure qPCR. All 32 upper respiratory samples analyzed from the COVID-19 cohort tested negative for MPXV as expected. No positive results were obtained when testing either respiratory or skin swab samples regardless of the Ag-RDT brand used 0% (95% CI 0%–20.59%). The specificity was 100% (95% CI 90.97%–100%) for the 3 Ag-RDT brands on both sample types (Table 3).

**Table 3:** Clinical diagnostic accuracy parameters of 3 MPXV antigen-detecting rapid diagnostic tests using retrospectively collected samples from 16 mpox patients and 32 COVID–19 patients, United Kingdom*

Category	Skin lesion swab samples, n = 30				Upper respiratory tract swab samples, n = 55		
	Ecotest	Flowflex	Standard Q		Ecotest	Flowflex	Standard Q
True positive	0	0	0		0	0	0
True negative	3	3	3		39	39	39
False positive	0	0	0		0	0	0
False negative	27	27	27		16	16	16
Specificity, % (95% CI)	100 (29.2–100)	100 (29.2–100)	100 (29.2–100)		100 (90.9–100)	100 (90.9–100)	100 (90.9–100)
Sensitivity, % (95% CI)	0 (0.0–12.7)	0 (0.0–12.7)	0 (0.0–12.7)		0 (0.0–20.6)	0 (0.0–20.6)	0 (0.0–20.6)
PPV, % (95% CI)	NA	NA	NA		NA	NA	NA
NPV, % (95% CI)	10.00 (3.5–25.6)	10.00 (3.5–25.6)	10.00 (3.5–25.6)		70.9 (57.9–81.2)	70.9 (57.9–81.2)	70.9 (57.9–81.2)
Sensitivity by Ct, % (95% CI)							
Ct <20	0 (0.0–84.2), n = 2	0 (0.0–84.2), n = 2	0 (0.0–84.2), n = 2		NA	NA	NA
Ct <25	0 (0.0–36.9), n = 8	0 (0.0–36.9), n = 8	0 (0.0–36.9), n = 8		0 (0.0–60.2), n = 4	0 (0.0–60.2), n = 4	0 (0.0–60.2), n = 4
Ct <33	0 (0.0–19.5), n = 17	0 (0.0–19.5), n = 17	0 (0.0–19.5), n = 17		0 (0.0–28.5), n = 11	0 (0.0–28.5), n = 11	0 (0.0–28.5), n = 11
Ct <40	0 (0.0–12.7), n = 27	0 (0.0–12.7), n = 27	0 (0.0–12.7), n = 27		0 (0.0–20.6), n = 16	0 (0.0–20.6), n = 16	0 (0.0–20.6), n = 16

*Tests evaluated: Ecotest, Ecotest Monkeypox Antigen Rapid Test (Assure Tech [Hangzhou] Co., Ltd., https://www.assuretech-product.com); Flowflex, FlowFlex Monkeypox Virus Antigen Rapid Test (ACON Biotech [Hangzhou] Co., Ltd., https://www.aconbio.com); Standard Q, Standard Q Monkeypox Ag Test (SD Biosensor, https://www.sdbiosensor.com). Ct, cycle threshold; MPXV, monkeypox virus; NA, not available; NPV, negative predictive value; PPV, positive predictive value.

**Figure 1:**
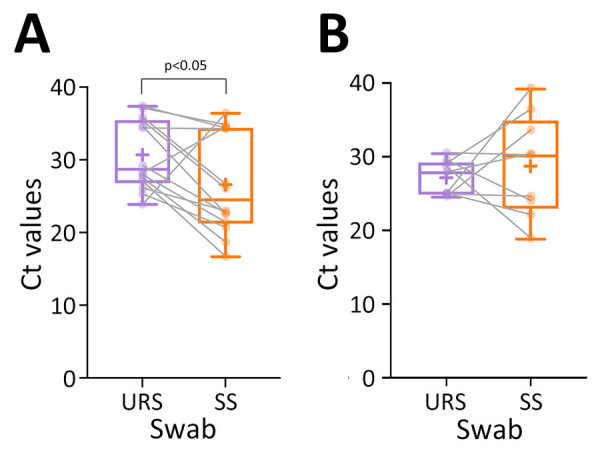
Boxplots of Ct values from paired URS and SS tested by Sansure quantitative PCR in study of diagnostic accuracy of 3 mpox lateral flow assays for antigen detection, Democratic Republic of the Congo (DRC) and United Kingdom. A) DRC, n = 14; B) United Kingdom, n = 9. Horizontal lines within boxes indicate medians, box tops and bottoms indicate interquartile range, and whiskers indicate maximum and minimum values. Ct values were significantly higher (p<0.05) in the URS group than in the SS group in the DRC cohort. Ct, cycle threshold; SS, skin lesion samples; URS, upper respiratory specimens.

**Figure 2:**
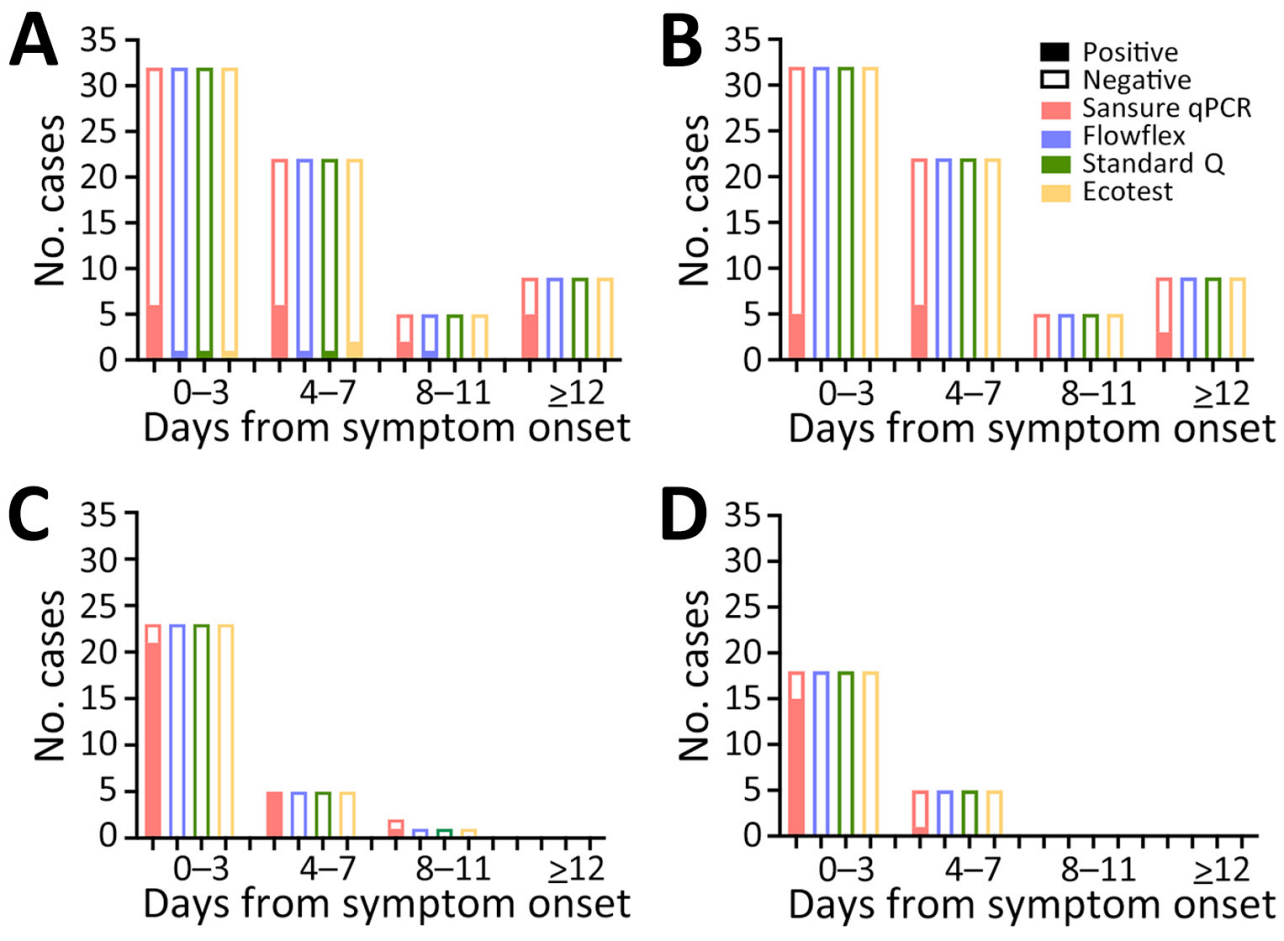
Number of positive and negative results by test and by days from symptom onset in study of diagnostic accuracy of 3 mpox lateral flow assays for antigen detection, Democratic Republic of the Congo (DRC) and United Kingdom. A) Skin lesion swab samples in DRC; B) upper respiratory swab samples in DRC; C) skin swab samples in the United Kingdom; D) upper respiratory swab samples in the United Kingdom. Rapid diagnostic tests evaluated: Ecotest, Ecotest Monkeypox Antigen Rapid Test (Assure Tech [Hangzhou] Co., Ltd., https://www.assuretech-product.com); Flowflex, FlowFlex Monkeypox Virus Antigen Rapid Test (ACON Biotech [Hangzhou] Co., Ltd., https://www.aconbio.com); Standard Q, Standard Q Monkeypox Ag Test (SD Biosensor, https://www.sdbiosensor.com). Sansure qPCR (Monkeypox Virus Nucleic Acid Diagnostic Kit; Sansure Biotech, https://www.sansureglobal.com) was used to evaluate results of the rapid diagnostic tests.

The difference in sensitivity in MPXV Ag-RDTs was lower when testing upper-respiratory samples than in skin samples (p = 0.007). We assessed the comparison of the Ct values and noted a difference in Ct values between upper-respiratory and skin sample groups (p=0.042) from DRC but not from the United Kingdom (Figure 1). The mean Ct value of upper-respiratory samples in DRC was 30.7 (+4.79) and mean Ct value for skin samples was 26.63 (+6.87), whereas in the United Kingdom mean Ct value for respiratory samples was 27.2 (+2.34) and for skin was 28.83 (+6.88). We found no difference in sensitivity between the 3 Ag-RDT brands and between countries. We also analyzed test results by onset of symptoms (Figure 2) but observed no difference in Ag-RDT results by symptom onset group.

### Analytical Evaluation

Using the viral stock, all Ag-RDT brands were positive at 5.0 × 10^4^ PFU/mL, 2.5 × 10^4^ PFU/mL, and 1.0 × 10^4^ PFU/mL. The LOD of all the Ag-RDT brands using the MPXV viral culture was determined to be 1.0 × 10^4^ PFU/mL (1.3 × 10^5^ copies/mL). All concentrations tested below the LOD were negative in all instances.

## Discussion

After the recent PHEIC, WHO issued an urgent call to accelerate availability of POC diagnostics for mpox (*17*). The lack of validation data for MPXV Ag-RDTs represents a large gap in the diagnostics landscape that has slowed down rapid, effective responses to new outbreaks and ongoing endemic transmission (*18*). The primary aim of this study was to evaluate the diagnostic accuracy of 3 Ag-RDT brands (Flowflex, Ecotest, and Standard Q) in DRC and the United Kingdom.

WHO’s target product profile (TPP) for MPXV Ag-RDTs recommends minimal clinical sensitivity of 80% and specificity of 97% (*19*). Specificity was fulfilled by the 3 Ag-RDTs evaluated in both countries, but sensitivity was extremely low (0%–15.79%), making the tests unsuitable for diagnostic or screening use. Evaluation data on Ag-RDT for MPXV are very limited. A previous study reported detection of MPXV antigens using the Ag-RDT Tetracore Orthopox BioThreat (https://tetracore.com) in 5 of 6 tested MPXV-positive samples with low Ct values (Ct 15–22) (*20*). In addition to the limited number of samples, this assay required sonication for swab material and dry ice/ethanol bath freezing followed by pestle grinding, making it unsuitable for POC use. Another study using an Orthopoxvirus Ag-RDT prototype failed to detect MPXV antigens among 80 MPXV qPCR-positive clinical samples in Belgium (*21*). That study suggested that the failure to detect MPXV antigen in swab samples could be caused by inhibition by the inactivating components of the VTM, which can cause protein denaturation. In this study, we used noninactivating swab transport medium in both sites and different types of medium (VTM in the DRC and UTM in the United Kingdom); clinical sensitivity was not improved. Studies on Ag-RDTs for SARS-CoV-2 comparing the use of dry swabs with Amies, VTM, and UTM have documented false-positive results because of nonspecific electrostatic interactions between the antibodies in the assay (*22*,*23*), a decreased LOD because of a dilution effect (*23*), or no changes in sensitivity or specificity depending on the Ag-RDT brand (*23*). The use of different types of swab transport medium should be investigated to optimize performance of Ag-RDT for mpox while preserving the virus for transport and storage.

Investigations of the analytical sensitivity of these Ag-RDTs gave an LOD of 1.0 × 10^4^ pfu/mL, being more sensitive than previous analytical evaluations of Ag-RDTs for MPXV. The Orthopoxvirus Ag-RDT prototype had an LOD of 3.0 × 10^5^ PFU/mL (*21*), and the commercially available Tetracore Orthopox BioThreat had an LOD of 1.5 × 10^6^ PFU/mL after sonication (*20*). The recommended analytical LOD in the WHO TPP is at 10^6^ PFU/mL, being fulfilled by the 3 brands of Ag-RDTs evaluated here and the previously published study on the Orthopoxvirus Ag-RDT prototype (*21*), suggesting that laboratory sensitivity using the PFU/mL measurement does not align with clinical sensitivity in the field.

The use of LOD using viral isolates is often used as a proxy before having the test evaluated using clinical specimens; however, in this study and others (*21*), the correlation between analytical and clinical sensitivity for MPXV has been shown to be very poor, yielding lower sensitivity than expected among clinical samples. The reason for this variability in antigen detection sensitivity between mpox clinical samples and mpox viral isolates is still uncertain and needs further investigation, as does the quantity and type of accessible antigen in clinical samples. In addition, the targeted antigen of the Ag-RDTs evaluated in this study was MPXV A29L. Target antigens for other Ag-RDT brands include A29L, A35R, A5L, B6R, E8L, H3, and M1R (*10*). The antigen A27L (homologous of MPXV A29L in vaccinia virus) has previously been suggested to be a good candidate because it is conserved and abundant within the virion; however, the Ag-RDTS targeting this antigen in this study and reported elsewhere (*21*) did not yield acceptable sensitivity. This finding highlights the need for further evaluations using clinical samples with Ag-RDTs that target different antigen types. Currently, manufacturers of >3 Ag-RDT brands in the market have disclosed the use of MPXV A29L as antigen target (Hangzhou Testsea Biotechnology, Guangdong Wesail Biotech, and Nanjing Synthgene Medical Technology).

Flowflex and Ecotest are designed to be used with upper-respiratory samples; however, none of the Ag-RDTs detected MPXV antigens in those samples, suggesting that using this sample type for antigen detection is not appropriate. Diagnostic evaluation studies using PCR found lower positivity rates in respiratory samples than in skin samples (*24*), which might be attributed to lower viral titer levels (*25*) or earlier clearance in this sample type (*26*), which is exacerbated by the lower sensitivity of the Ag-RDTs evaluated here.

Major limitations of this study were that testing could not be done at the POC in DRC (because samples had to be transported to the designated laboratory) and that retrospective frozen samples were used in the United Kingdom. The effect of testing delay and of sample storage and freeze-thawing on Ag-RDT results has not been studied with MPXV. Studies on SARS-CoV-2 Ag-RDTs noted a decline in test-line intensity (not false-negative results) after storage periods of 24 hours to >7 days at 2°C–8°C (*27*–*30*). Results of Ag-RDT testing for SARS-CoV-2 did not find a significant difference between 101 datasets that involved fresh specimens and 23 freeze-thawed specimens (*31*). However, MPXV is a larger DNA virus, whereas SARS-CoV-2 is a smaller RNA virus; thus, the 2 are not directly comparable. The use of retrospective frozen and refrigerated samples is accepted for production of clinical diagnostic data (*13*), and the WHO TPP recommends that MPXV Ag-RDTs be compatible with samples that have been refrigerated or frozen with use of preservation media for quality control, repeats, or follow-up testing (*1*9).

In conclusion, the results of this study raise considerable doubts on the suitability of Ag-RDT for mpox surveillance and diagnosis because of their poor clinical sensitivity among suspected mpox cases. Recommendations for future mpox Ag-RDT evaluations should include brands that detect different MPXV antigens and evaluation of different swab preservation mediums.

This article was published as a preprint at https://www.medrxiv.org/content/10.1101/2024.11.07.24316894v1.
